# The associations between body fat distribution and bone mineral density in the Oxford Biobank: a cross sectional study

**DOI:** 10.1080/17446651.2022.2008238

**Published:** 2021-12-03

**Authors:** Catriona Hilton, Senthil K Vasan, Matt J Neville, Constantinos Christodoulides, Fredrik Karpe

**Affiliations:** a Oxford Centre for Diabetes, Endocrinology and Metabolism, Radcliffe Department of Medicine, University of Oxford, Churchill Hospital, Oxford, UK; b NIHR Oxford Biomedical Research Centre, OUH Trust, Oxford, UK

**Keywords:** Obesity, bone mineral density, body fat distribution, dual energy x-ray absorptiometry, lean mass

## Abstract

**Background:**

Body composition is associated with bone mineral density (BMD), but the precise associations between body fat distribution and BMD remain unclear. The regional adipose tissue depots have different metabolic profiles. We hypothesized that they would have independent associations with BMD.

**Research Design and Methods:**

We used data from 4,900 healthy individuals aged 30–50 years old from the Oxford Biobank to analyze associations between regional fat mass, lean mass and total BMD.

**Results:**

Total lean mass was strongly positively associated with BMD. An increase in total BMD was observed with increasing mass of all the fat depots, as measured either by anthropometry or DXA, when accounting for lean mass. However, on adjustment for both total fat mass and lean mass, fat depot specific associations emerged. Increased android and visceral adipose tissue mass in men, and increased visceral adipose tissue mass in women, were associated with lower BMD.

**Conclusions:**

Fat distribution alters the association between adiposity and BMD.

## 1. Introduction

Loss of bone mineral density (BMD) leading to osteoporosis or osteopenia causes significant morbidity [1]. These are complex disorders impacted on by aging [2], sex hormones [1], genetic predisposition [3], physical activity/lean body mass [2], but also by adiposity through the concept of ‘sarcopenic obesity’ [2]. Obesity, and in particular upper body fat [4], impacts the metabolic environment adversely. Obesity and low BMD are common. Nearly a third of the world’s population is affected by overweight or obesity [5]. Osteoporosis affects 2% of 50-year-olds, with prevalence rising to more than 25% in women aged 80 and over [6]. Despite this, the relationships between body fat mass, regional fat distribution and bone mineral density are not well understood. A greater understanding of how body composition influences BMD will help identify populations at greater risk of osteoporosis and inform public health measures.

Adipose tissue (AT) is not a homogenous organ but is composed of several fat depots which are distinct in terms of their structural, functional, and metabolic properties, as well as developmental origin (reviewed here [4]). The distribution of body fat has a causative impact on metabolic health. Central body fat increases the risk of type 2 diabetes and cardiometabolic disease, whilst lower body adiposity is protective [7].

Bone remodels in response to the mechanical stress placed on it, both by weight bearing through the bone and by muscle contraction [8]. A positive association has consistently been demonstrated between lean mass and BMD [9–12]. Several studies have shown that BMD increases with increased total AT mass [9,10,13] but absence of association has also been described [12]. The current literature on the associations between body fat distribution and BMD is conflicting: whilst some groups have observed an inverse correlation between abdominal fat deposition and BMD [14–17] others have failed to find an association [18] and a positive correlation has also been reported [19,20]. Abdominal fat can be sub-divided into the subcutaneous abdominal and visceral compartments. The relationship between visceral adipose tissue (VAT) mass and BMD is also unclear, with both a negative association [10,11,21,22] and lack of association [18,23] having been described.

Some of the disparity in the literature on body fat distribution and BMD is likely to relate to the populations studied, as both fat distribution and BMD are influenced by ethnicity, sex [24], age, and menopausal status [25–27]. Study designs vary, with some studies being underpowered or failing to isolate the contribution of regional fat depots independent of total body fat or lean mass. Study findings can also be influenced by varying statistical approaches.

We have previously shown that gain of function *LRP5* mutations leading to high bone mass are also associated with increased lower body fat accumulation [28]. This observation points toward possible shared pathways for regional tissue expansion and BMD. We therefore hypothesized that the individual AT depots, independent of total fat mass, would display potentially disparate associations with BMD. Beyond this, we recognize that regional fat depots have independent relationships with whole-body metabolic features such as insulin resistance, and this was also taken into account. In this study we delineated the associations between body fat distribution and BMD using data from the Oxford Biobank (OBB), a unique cohort with detailed dual-energy X-ray absorptiometry (DXA) characterization of regional AT depots, lean mass and BMD for nearly 5,000 healthy men and women [29].

## 2. Patients and methods

### 2.1. Participants and study methods

The OBB includes more than 8,000 randomly recruited population-based Caucasian men and women aged between 30 and 50 residing in Oxfordshire [29]. Pregnant women and individuals with previous diagnoses of myocardial infarction or heart failure currently on treatment, untreated malignancies, diabetes or other systemic ongoing disease are excluded from participation. Nearly 5,000 participants have undergone DXA scans for determination of BMD and body fat distribution. The characteristics of the study population are described in Table 1. Information on physical activity, smoking, and alcohol intake was obtained using validated questionnaires. Physical activity levels were categorized as sedentary, moderate activity, active and fit based on their engagement with exercise at home and work. Smoking status was stratified as never smoker, ex-smoker, and current smoker. Alcohol intake was based on the number of units of alcohol consumed per week and was categorized as excessive (>21 units/week for men or >14 units/week for women) or limited (within recommended limits). Venous blood samples were taken after an overnight fast. Insulin resistance was estimated using the homeostasis model assessment (HOMA IR) according to the formula: fasting insulin (microU/L) x fasting glucose (nmol/L)/22.5 [30]. For the purpose of this study, obesity was defined as BMI > 30 kg/m^2^.

**Table 1.** Characteristics of the study population

	Men	Women
	(n = 2,101)	(n = 2,805)
Age (years)	41.4 ± 5.9	41.2 ± 6.0
Non-smokers†	1,269 (60.5)	1,708 (60.9)
*Alcohol status†a*		
Nondrinkers	16 (0.76)	86 (3.07)
Moderate drinkers	1,820 (86.8)	2,483 (88.6)
Heavy drinkers	261 (12.5)	234 (8.4)
*Physical activity†^b^*		
Sedentary	89 (4.2)	121 (4.3)
Moderate intensity	1,178 (56.2)	1,973 (70.3)
Heavy intensity	830 (39.6)	709 (25.3)
*Anthropometry*		
Height (cm)	179.2 ± 6.5	165.7 ± 6.3
Weight (cm)	85.6 ± 14	69.4 ± 13.6
BMI (kg/m^2^)	26.6 ± 4.0	25.3 ± 4.8
Waist circumference (cm)	93.1 ± 11.1	82.5 ± 12.3
Hip circumference (cm)	101.8 ± 7.2	101.4 ± 9.7
*Body composition by DXA*		
Android fat (kg)^‡^	2.07 (1.4, 2.9)	1.61 (1.0, 2.4)
Visceral fat (kg) ^‡^	1.0 (0.5, 1.6)	0.3 (0.1, 0.6)
Gynoid fat (kg) ^‡^	3.3 (2.5, 4.1)	4.3 (3.4, 5.4)
Leg fat (kg) ^‡^	6.1 (4.8, 7.5)	8.4 (6.9, 10.7)
Total lean mass (kg) ^‡^	57.8 (53.6, 62.5)	41.1 (37.9, 44.5)
Total BMD (g/cm^2^) ^‡^	1.27 (1.20, 1.34)	1.17 (1.11, 1.25)
Pelvic BMD (g/cm^2^) ^‡^	1.12 (1.04, 1.22)	1.06 (0.98, 1.15)
Spine BMD (g/cm^2^) ^‡^	1.17 (1.08, 1.26)	1.10 (1.02, 1.19)
Arms BMD (g/cm^2^) ^‡^	0.89 (0.82, 0.98)	0.77 (0.71, 0.84)
Legs BMD (g/cm^2^) ^‡^	1.39 (1.31, 1.48)	1.20 (1.13, 1.27)
HOMA IR^‡^	2.9 (2.2, 4.0)	2.4 (1.8, 3.3)
Obesity (BMI>30)†	367 (17.4)	427 (15.2)
Post-menopausal†	-	198 (7.5)

Data presented as mean ± standard deviation and **^‡^**Median (inter-quartile range) for continuous variables; **^†^**frequency (percentage) for categorical variables.

aAlcohol intake: moderate consumption, less than 21 units in men and less than 14 units in women (per week); heavy consumption, greater than 21 units in men and greater than 14 units in women (per week). bPhysical activity classified as moderate and vigorous activity per week.

DXA scans were performed using the Lunar iDXA (GE Healthcare, Madison, WI, USA) and images were processed using enCORE v14.1 software (GE Healthcare, Madison, WI, USA). All DXA scans were performed on the same machine. Bone mineral calibration and quality control were performed using a spine phantom according to the manufacturer’s instructions. The android region is defined by the iliac crest at the lower boundary and the upper boundary is calculated as 20% of the distance between the neck and the iliac crest. The gynoid region includes the upper thighs and hips. It is twice the height of the android region with the upper boundary located below the iliac crest by 1.5 times the height of the android region. VAT is calculated by the enCORE v14.1 software using a predefined algorithm [31]. Subregional BMD was calculated using data from a total body DXA scan, analyzed using manufacturer recommended methodology for the delineation of subregions.

Ethical approval was granted by Oxfordshire Clinical Research Ethics Committee (08/H0606/107) and study participants provided written informed consent.

### 2.2. Statistical methods

Descriptive data are summarized as mean and standard deviation (SD) for normally distributed variables and median and interquartile range (IQR) for skewed variables. Categorical data are presented as frequency and percentages. Pearsons’ correlation coefficient was used to examine the relationship between various fat depots, lean mass and bone mass (Table 2). We generated age- and sex-specific z-scores for DXA-measured fat depots and for waist and hip circumference using Fisher-Yates transformation [32], to allow direct comparison of risk magnitude per 1 standard deviation (SD) change. Z-transformed exposures were used in linear regression models to examine the association between total BMD, fat and lean mass and estimates are presented as standardized beta (sß). Sex-stratified and BMI-based stratification models are presented: model 1: adjusted for confounders such as age, height, smoking status, alcohol intake, physical activity and menopausal status in women; model 2: adjusted for total fat mass in addition to the covariates as above and model 3: adjusted for HOMA IR [30] in addition to the covariates as in model 2. Additionally, the effect of regional fat depots (android, VAT, gynoid and leg) on total, spine, pelvic, arm and leg BMD were examined using linear regression models adjusted for age and total lean mass (model 1), mutually adjusted for age, total lean and total fat mass (model 2) and mutually adjusted for age, total lean mass, total fat mass and HOMA IR (model 3). To test for multi-collinearity between closely related fat depots, we calculated variable inflation factor (VIF) for each regression model (Table S1). A VIF of <5 was considered as absence of collinearity. All analyses were performed using STATA Version 13.1 (College Station, Texas, USA).

**Table 2.** Correlation matrix for DEXA-derived adipose tissue and bone variables

	BMD-Spine	BMD-pelvis	Android fat	Gynoid fat	Total fat	Visceral fat	Total lean mass
BMD-Spine	1						
BMD-pelvis	0.8177	1					
Android fat	0.3514	0.1694	1				
Gynoid fat	0.1891	0.0374	0.7392	1			
Total fat	0.3048	0.1218	0.9417	0.9073	1		
Visceral fat	0.2501	0.1312	0.7033	0.3003	0.554	1	
Total lean mass	0.4659	0.4478	0.3684	−0.0579	0.1948	0.4645	1

## 3. Results

### 3.1. Cohort characteristics

The study cohort included 2,101 men (43%) and 2,805 women, with a mean age of 41 years. Most participants were moderately physically active based on activity questionnaires, 39.3% smoked and 7.5% of women reported they were post-menopausal at the time of recruitment. As expected, women had a gynoid distribution of body fat in comparison to the predominantly android fat distribution seen in men. BMD was comparable in men and women. Detailed cohort characteristics are shown in Table 1.

### 3.2. Total lean mass, total fat mass, and total bone mineral density

A strong positive correlation was observed between total and regional fat masses, particularly android and gynoid fat mass, as shown in Table 3. Correlations between fat mass and bone or lean mass were relatively weaker.

**Table 3.** Association of total fat and total lean mass with total BMD in individuals with and without obesity

	Total cohort			Without obesity (BMI<30 kg/m^2^)			With obesity (BMI>30 kg/m^2^)		
	N	sβ	p-value	N	sβ	p-value	N	sβ	p-value
Men (n = 2,097)									
Lean mass	2097	0.57	1.51 × 10^−126^	732	0.56	3.52 × 10^−84^	365	0.45	3.00 × 10^−11^
Fat mass	2097	0.29	4.63 × 10^−40^	1732	0.19	8.41 × 10^−15^	365	0.02	0.78
Fat mass adj lean mass	2097	0.10	1.33 × 10^−05^	1732	0.13	8.50 × 10^−09^	365	0.01	0.91
Fat mass adj HOMA IR*	2097	0.14	3.01 × 10^−10^	1732	0.18	1.09 × 10^−14^	365	0.07	0.18
Lean mass adj fat mass	2097	0.51	2.45 × 10^−25^	1732	0.49	6.01 × 10^−16^	365	0.46	1.42 × 10^−19^
Women (n = 2,802)									
Lean mass	2802	0.49	1.16 × 10^−113^	2376	0.44	5.49 × 10^−63^	426	0.37	7.01 × 10^−09^
Fat mass	2802	0.31	1.10 × 10^−58^	2376	0.20	9.53 × 10^−22^	426	−0.03	0.66
Fat mass adj lean mass	2802	0.13	4.92 × 10^−11^	2376	0.14	1.13 × 10^−11^	426	−0.13	0.015
Fat mass adj HOMA IR*	2802	0.17	1.84 × 10^−15^	2376	0.17	1.31 × 10^−15^	426	−0.11	0.043
Lean mass adj fat mass	2802	0.40	1.25 × 10^−98^	2376	0.38	8.02 × 10^–84^	426	0.43	2.09 × 10^−16^

Sβ represents corresponding SD increase in total BMD with one SD increase in fat and lean mass. Data presented for z-transformed fat and lean mass. All linear regression models adjusted additionally for age, height, smoking status, alcohol intake, physical activity and menopausal status in women.

*fat mass, lean mass and HOMA IR adjusted.

We observed a strong positive association between total lean mass and total BMD (men: sβ = 0.571, p = 1.51×10^−126^; women: sβ = 0.492, p = 1.16×10^−113^; Table 3) in individuals both with and without obesity. In people without obesity (BMI < 30 kg/m^2^), total fat mass was also positively associated with total BMD (men: sβ = 0.189, p = 8.41×10^−15^; women: sβ = 0.199, p = 9.53 × 10^−22^). Adjustment for total lean mass attenuated but did not abolish the association between total fat mass and total BMD in people without obesity (men: sβ = 0.128, p = 8.50×10^−09^; women: sβ = 0.137, p = 1.13×10^−11^), suggesting an independent and positive effect of adipose tissue on BMD. Adjustment for HOMA IR did not change these findings.

The presence of obesity did not substantially alter the associations between total lean mass and total BMD. However, in men with obesity (BMI > 30 kg/m^2^) the positive association between total BMD and total fat mass accounted for total lean mass was lost (sβ = 0.007, p = 0.91). In women with obesity total fat mass when accounted for total lean mass was negatively associated with BMD (sβ = −0.133, p = 0.015), i.e. the association was reversed, and this association remained significant after adjustment for HOMA IR (sβ = −0.112, p = 0.043).

### 3.3. Associations between regional adiposity and total BMD

Having established an independent association between total fat mass and total BMD, we went on to investigate the effect of body fat distribution (Table 4). A significant positive association was observed between all regional AT depots, except VAT, and total BMD when adjusted for lean mass.

**Table 4.** Association between total BMD and regional adiposity measured using anthropometry and DXA

		Model 1	Model 2	Model3
		Sβ (p value)	Sβ (p value)	Sβ (p value)
	Total BMD			
	z-waist	0.059 (0.016)	−0.161 (0.001)	−0.113 (0.021)
	z-hip	0.103 (2.23 × 10^−05^)	0.057 (NS, 0.15)	0.043 (NS, 0.27)
Men (n = 2,097)	z-android	0.072 (0.001)	−0.360 (7.80 × 10^−05^)	−0.171 (NS, 0.06)
	z-VAT	0.034 (NS, 0.13)	−0.144 (2.41 × 10^−04^)	−0.076 (NS, 0.06)
	z-gynoid	0.091 (1.91 × 10^−05^)	0.033 (NS, 0.59)	−0.019 (NS, 0.75)
	z-leg	0.109 (6.07 × 10^−07^)	0.116 (0.014)	0.021 (NS, 0.65)
	z-waist	0.109 (8.66 ×10^−07^)	−0.013 (NS, 0.71)	0.006 (NS, 0.85)
	z-hip	0.144 (1.04 × 10^−11^)	0.072 (NS, 0.074)	0.054 (NS, 0.17)
Women (n = 2,658)	z-android	0.132 (5.80 × 10^−10^)	0.058 (NS, 0.35)	0.144 (0.032)
	z-VAT	0.023 (NS, 0.25)	−0.151 (1.72 × 10^−07^)	−0.118 (6.21 × 10^−05^)
	z-gynoid	0.106 (2.98 × 10^−08^)	−0.025 (NS, 0.64)	−0.067 (NS, 0.21)
	z-leg	0.108 (3.06 × 10^−09^)	−0.023 (NS, 0.61)	−0.082 (NS, 0.06)

Sβ represents corresponding SD increase in total BMD with one SD increase in regional fat measured using DXA and anthropometry and total lean mass. Data presented for z-transformed fat and lean mass.

Model 1: adjusted for total lean mass, age, height, smoking status, alcohol intake, physical activity and menopausal status in women.

Model 2: Model 1 + adjusted additionally for total fat mass.

Model 3: Model 2 + adjusted additionally for HOMA IR.

However, when further accounting for total fat mass (in order to isolate associations with individual AT depots) opposite associations were seen between VAT and total BMD in both sexes (men: sβ = −0.144, p = 2.41×10^−04^; women: sβ = −0.151, p = 1.72 × 10^−07^; Table 4). For men, waist circumference and total android fat mass also displayed similar negative associations with total BMD (Table 4). These associations were lost when additionally adjusted for HOMA IR, except for waist circumference which attenuated, yet remained significant (sβ = −0.113, p = 0.021). In men, a strong collinearity was observed between total fat mass and android fat mass (VIF > 5). Other regional fat depots did not demonstrate collinearity with total fat mass.

In contrast, there was a significant positive association between leg AT and total BMD in men; following adjustment for total fat mass, one SD increase in leg fat was associated with 0.116 SD increase in total BMD (p = 0.014), which was directionally opposite to the estimates observed with central adipose depots. These opposing associations were lost following adjustment for HOMA IR.

### 3.4. Associations between regional adiposity and regional BMD

The associations between regional BMD and the regional fat depots are shown in Table S1. Higher VAT mass in women was associated with significant reduction in pelvic, arm and leg BMD (Model 2, Table S1). In men, higher android fat and VAT mass were associated with significantly lower BMD of the arm (android fat: sβ = −0.025, p = 1.67 × 10–09; VAT; sβ = −0.019, p = 0.0003).

## 4. Discussion

### 4.1. Skeletal muscle and BMD

Consistent with previous studies [9–12] we found a strong positive association between lean mass and BMD. Skeletal muscle is important for stimulating bone remodeling both by direct load bearing and by placing mechanical strain across bone [8]. In addition, skeletal muscle and bone influence one another via cross-talk between their secretomes [33]; for example, prostaglandin E2 secreted from bone enhances myogenesis [34] and myostatin, a myokine which negatively regulates muscle growth, preserves bone density [35].

### 4.2. Obesity and BMD

In people not affected by obesity, fat mass was positively associated with BMD independent of lean mass. Conversely, in men with obesity the positive association between increasing AT mass and BMD was lost, and in women reversed, suggesting that with obesity the detrimental actions of AT on bone may begin to outweigh its protective effects. Similarly, in a study of an older (45–67-year-old) Caucasian population, Zhu e*t al* [36] observed that for women, but not for men, higher fat mass for BMI was associated with a lower BMD.

### 4.3. Body fat distribution and BMD

Regional adiposity is related to total fat mass, and so we investigated the degree of multicollinearity between variables. High collinearity was observed in models where android fat mass and total fat mass were included, with this effect being stronger for men. This indicates that total fat mass might be driving associations between android fat mass and BMD. We observed a negative association between VAT mass and total BMD after adjustment for lean mass and total fat mass. This is consistent with the growing body of evidence that central obesity is more damaging to health than lower body obesity [37].

In line with our findings that VAT was negatively associated with BMD, one group assessed bone microarchitecture of trans-iliac bone biopsies from women who were premenopausal and found that increased trunk fat was associated with inferior bone quality and lower rates of bone formation (although the latter effect disappeared after correction for BMI) [38]. VAT mass has also been negatively linked to bone mechanical properties in men with obesity [39]. Furthermore, whilst rapid weight loss following sleeve gastrectomy is associated with loss of BMD, fat loss from the VAT depot appears to protect against this [40].

The observation that VAT and total adiposity have opposite relationships to BMD implies that AT exerts effects on bone beyond simple load bearing. This is supported by our finding that the negative associations between VAT and BMD are consistent for upper and lower body bone. In our cohort, adjustment for insulin resistance as estimated by HOMA IR attenuated the negative association between VAT mass, but not total AT mass, and BMD. Insulin resistance may have a causative role in the detrimental effect of visceral adiposity on bone metabolism. Insulin itself has been shown to have anabolic effects on bone metabolism [41] and so the mechanism by which insulin resistance impacts on BMD may be through associated factors (discussed below).

### 4.4. Mechanisms linking body fat and BMD

#### 4.4.1. Mechanisms in both sexes

There are several other plausible mechanisms by which AT mass and distribution and bone metabolism might be linked. These include effects of AT-derived factors on bone, effects of bone-derived factors on AT, and common drivers of bone and AT development and metabolism.

Bone mineralization has been suggested to be positively modulated by AT through the direct effect of weight loading and indirectly through circulating insulin [41], adipokines [42], and increased aromatization of androgens [43]. Furthermore, leptin [44] and adiponectin [45,46] both act centrally on the sympathetic nervous system to regulate bone mass. In obesity the balance can shift so that some adipose associated factors have a negative, rather than positive, effect on bone metabolism. For example, obesity in men [47], and in particular central obesity [48], is associated with lower testosterone levels. Conversely, increased AT mass is also correlated with a more inflammatory profile of circulating cytokines [49]. Many of these, including TNFa, the interleukin family (IL-1, IL-12, IL-17, IL-18, and IL-33) and interferons, directly decrease bone formation or increase bone resorption [50]. Relevant to our findings, abdominal, and particularly visceral, adiposity is associated with a more inflammatory adipokine profile than lower body fat [51]. Dietary factors may also play a role. A high fat diet has been postulated to cause bone loss [52]. Vitamin D deficiency and secondary hyperparathyroidism are both well documented to affect bone health and are more common in people with obesity. Reciprocally, bone-derived factors can also influence adipogenesis [53].

Several systemic factors are known to control both adipogenesis and skeletal health. Sex hormones are discussed below. Sympathetic tone increases energy expenditure by increasing lipolysis [54] and may inhibit pre-adipocyte proliferation [55] as well as directly inhibiting bone turnover and reducing bone mineral density [56]. Finally, it could also be hypothesized that there is an underlying genetic influence on bone, muscle and AT development, such that in lean individuals BMD is proportional to total adipocyte and myocyte number. This relationship could be disrupted in obesity, where adipocyte number, as well as adipocyte size, can increase. Twin studies have suggested that BMD may share genetic determinants with lean mass and, to a lesser extent, fat mass [57]. Several common genetic drivers for bone mineral density and total body fat mass and body fat distribution have been identified: a number of signals associated with BMD in genome-wide association studies (GWAS) have also been found to associate with obesity phenotypes [3,58] and it has been observed that the obesity-linked variant FTO is also associated with reduced BMD [59].

#### 4.4.2. The role of sex hormones

We observed a sexual dichotomy in the relationship between adiposity and BMD, consistent with the findings of other groups [10]. Both estrogen and testosterone have anabolic effects on bone [60].

Sex hormones play an important role in determining both fat mass and distribution and skeletal maturation and turnover. In men, androgens protect against fat accumulation, particularly in the visceral compartment [47,48]. Conversely, in women, androgen excess predisposes to central obesity [61]. Estrogens inhibit fat accrual in both men and women through effects on energy intake and expenditure as well as local effects in AT [62]. However, in men the relationship between estrogens and adiposity is complex. In men, estrogens are primarily produced by peripheral aromatization of androgens, including in AT [62] and androgen aromatization rate increases with fat mass [63]. Male obesity is associated with an increase in estrogen levels, which may then negatively feedback to further reduce androgen production [63].

### 4.5. Limitations and areas for further research

A detailed dissection of the factors by which bone metabolism and AT might be linked was beyond the scope of this study. Further research will be required to investigate the mechanisms regulating the associations between body fat distribution and BMD more fully. Although some of the associations with regional fat measurements and BMD emerged as significant, we acknowledge the issue of multi-collinearity of regional and total fat mass, and these effects may be either fully or partially driven by total fat mass. Due to the nature of our data, we are unable to speculate on the direction of causality, and future longitudinal studies will be required to determine the relative contribution of AT on bone metabolism, bone on AT metabolism and shared genetic and developmental drivers. We were limited by the data available for this cohort and so were unable to include variables such as sex hormones, vitamin D, PTH and dietary calcium and vitamin D intake.

It should be noted that our cohort included healthy young (30–50 year old) Caucasian men and women, and that although a number of women were post- or peri-menopausal the majority were pre-menopausal. Physical activity is an important determinant of BMD, and we were limited to accounting for this according to self-reported activity levels. It should also be acknowledged that the BMD regions defined in this study are different to those used in clinical determination of osteoporosis risk. Our DEXA data did not include trabecular bone score, which would have given valuable information on the effect of body fat distribution on bone microarchitecture. Furthermore, although BMD is strongly associated with fracture risk it only accounts for a component of overall risk, with most fractures occurring in non-osteoporotic individuals [64].

## 5. Conclusions

These data support the hypothesis that body fat distribution, in addition to total adiposity, is important to determining bone mineral density. Insulin resistance may be a mechanism by which VAT negatively modulates bone metabolism. These findings will aid in the recognition of people with obesity most at risk of osteoporosis, and add to the evidence that central obesity is more harmful to health than lower body obesity [4]. Further research will be required to elucidate the clinical relevance of this observation and the mechanisms involved.

## Supplementary Material

Supplemental Material
