# Supplementary material for: The associations between body fat distribution and bone mineral density in the Oxford Biobank: a cross sectional study
Source: Expert Rev Endocrinol Metab. 2021 Dec 3;17(1):75–81. doi: 10.1080/17446651.2022.2008238 (PMC8944227; doi:10.1080/17446651.2022.2008238)
Supplement: Supplemental Material [file IERE_A_2008238_SM8158.docx]

**Supporting information**

**Supplementary Table 1: Association between regional adipose depots and regional BMD.** Variable inflation factors (VIF) are shown for regression models. A VIF of <5 was considered acceptable indicating no collinearity between the adjusted variables.

|  |  | Unadjusted model | Model adjusted for age, | **Model 1** |  | **Model 2** |  | **Model3** |  |
| --- | --- | --- | --- | --- | --- | --- | --- | --- | --- |
|  |  |  | height, smoking, PA | Total lean mass + age | VIF | Model 1+ total fat |  | Model 2+ HOMA- |  |
|  |  |  | (menopausal status in women | adjusted |  | mass adjusted | VIF | -IR adjusted | VIF |
|  |  |  | only) | Sβ (p value) |  | Sβ (p value) |  | Sβ (p value) |  |
|  | **Pelvis BMD** |  |  |  |  |  |  |  |  |
| **Men (n=2,097)** | z-waist | 0.175 (5.53 x 10^-16^) | 0.194 (5.67 x 10^-19^) | -0.038 (1.59 x 10^-06^) | 1.42 | -0.028 (0.0002) | 2.67 | -0.026 (0.006) | 2.54 |
|  | z-hip | 0.278 (1.42 x 10^-38^) | 0.276 (4.49 x 10^-35^) | 0.011 (NS)) | 1.45 | 0.019 (NS) | 2.01 | 0.018 (NS) | 1.97 |
|  | z-android | 0.105 (1.42 x 10^-06^) | 0.146 (3.68 x 10^-11^) | -0.016 (NS) | 1.29 | -0.002 (NS) | **7.11** | -0.0004 (NS) | **6.59** |
|  | z-VAT | 0.064 (0.004) | 0.106 (1.11 x 10-06) | -0.013 (NS) | 1.31 | -0.003 (NS) | 1.74 | 0.001 (NS) | 1.73 |
|  | z-gynoid | 0.117 (0.52 x 10^-08^) | 0.142 (2.34 x 10^-10^) | -0.008 (NS) | 1.25 | 0.005 (NS) | 3.87 | 0.003 (NS) | 3.68 |
|  | z-leg | 0.093 (0.00001) | 0.112 (5.24 x 10^-07^) | -0.026 (NS) | 1.23 | -0.013 (NS) | 2.72 | -0.017 (NS) | 2.68 |
|  |  |  |  |  |  |  |  |  |  |
| **Women (n=2,658)** | z-waist | 0.203 (3.73 x 10^-26^) | 0.230 (1.42 x 10^-31^) | 0.035 (NS) | 1.33 | 0.016 (NS) | 1.74 | 0.008 (NS) | 1.71 |
|  | z-hip | 0.257 (7.46 x 10^-44^) | 0.249 (6.46 x 10^-39^) | 0.027 (NS) | 1.37 | -0.002 (NS) | 2.15 | -0.002 (NS) | 2.11 |
|  | z-android | 0.179 (9.03 x 10^-22^) | 0.219 (1.54 x 10^-29^) | 0.057 (NS) | 1.27 | 0.021 (NS) | 4.39 | 0.022 (NS) | 4.18 |
|  | z-VAT | 0.103 (3.99 x 10^-08^) | 0.131 (8.27 x 10^-12^) | -0.012 (NS) | 1.24 | -0.041 (0.002) | 1.35 | -0.037 (0.003) | 1.38 |
|  | z-gynoid | 0.178 (1.86 x 10^-21^) | 0.184 (1.67 x 10^-21^) | 0.021 (<0.001) | 1.25 | -0.013 (NS) | 3.25 | -0.015 (NS) | 3.16 |
|  | z-leg | 0.150 (1.29 x 10^-15^) | 0.146 (3.56 x 10^-14^) | -0.014 (NS) | 1.24 | -0.047 (NS) | 2.35 | -0.049 (NS) | 2.38 |
|  | **Spine BMD** |  |  |  |  |  |  |  |  |
| **Men (n=2,097)** | z-waist | 0.371 (2.45 x 10^-69^) | 0.388 (4.85 x 10^-73^) | 0.123 (NS) | 1.42 | -0.012 (NS) | 2.67 | -0.009 (NS) | 2.54 |
|  | z-hip | 0.425 (5.01 x 10^-93^) | 0.433 (1.63 x 10^-86^) | 0.129 (NS) | 1.45 | 0.019 (NS) | 2.01 | 0.017 (NS) | 1.97 |
|  | z-android | 0.308 (2.20 x 10^-47^) | 0.346 (8.95 x 10^-57^) | 0.169 (NS) | 1.29 | 0.002 (NS) | **7.11** | 0.005 (NS) | **6.59** |
|  | z-VAT | 0.253 (3.38 x 10^-32^) | 0.291 (1.38 x 10^-40^) | 0.156 (NS) | 1.31 | 0.018 (NS) | 1.74 | 0.025 (NS) | 1.73 |
|  | z-gynoid | 0.282 (9.42 x 10^-40^) | 0.306 (7.39 x 10^-43^) | 0.146 (NS) | 1.25 | -0.020 (NS) | 3.87 | -0.023 (NS) | 3.68 |
|  | z-leg | 0.262 (2.65 x 10^-34^) | 0.279 (3.88 x 10^-36^) | 0.142 (NS) | 1.23 | -0.021 (NS) | 2.72 | -0.027 (NS) | 2.68 |
|  |  |  |  |  |  |  |  |  |  |
| **Women (n=2,658)** | z-waist | 0.350 (2.23 x10^-77^) | 0.361 (5.53 x 10^-77^) | 0.189 (1.47 x 10^-21^) | 1.33 | -0.022 (NS) | 1.74 | 0.027 (0.0007) | 1.71 |
|  | z-hip | 0.410 (3.46 x 10^-44^) | 0.403 (6.28 x 10^-38^) | 0.191 (NS) | 1.37 | 0.017 (NS) | 2.15 | 0.016 (NS) | 2.11 |
|  | z-android | 0.361 (2.70 x 10^-87^) | 0.388 (1.43 x 10^-92^) | 0.244 (NS) | 1.27 | 0.031 (NS) | 4.39 | 0.035 (4.42 x 10^-09^) | 4.18 |
|  | z-VAT | 0.219 (7.99 x 10^-32^) | 0.231 (1.60 x 10^-33^) | 0.189 (NS) | 1.24 | 0.023 (NS) | 1.35 | 0.034 (NS) | 1.38 |
|  | z-gynoid | 0.335 (6.35 x 10^-75^) | 0.341 (2.78 x 10^-71^) | 0.186 (NS) | 1.25 | -0.021 (NS) | 3.25 | -0.025 (NS) | 3.16 |
|  | z-leg | 0.303 (6.10 x 10^-61^) | 0.302 (1.01 x 10^-55^) | 0.149 (NS) | 1.26 | -0.051 (NS) | 2.35 | -0.06 (NS) | 2.38 |
|  | **Arm BMD** |  |  |  |  |  |  |  |  |
| **Men (n=2,097)** | z-waist | 0.266 (2.48 x 10-35) | 0.282 (2.43 x 10^-37^) | 0.066 (NS) | 1.42 | -0.009 (NS) | 2.67 | -0.004 (NS) | 2.54 |
|  | z-hip | 0.296 (8.82 x 10^-44^) | 0.304 (8.68 x 10^-41^) | 0.056 (NS) | 1.45 | -0.006 (NS) | 2.01 | -0.011 (NS) | 1.97 |
|  | z-android | 0.194 (2.34 x 10^-19^) | 0.226 (7.27 x 10^-24^) | 0.070 (NS) | 1.29 | -0.025 (1.67 x 10^-09^) | **7.11** | -0.018 (2.14 x 10^-06^) | **6.59** |
|  | z-VAT | 0.159 (2.29 x 10^-13^) | 0.188 (2.67 x 10^-17^) | 0.044 (NS) | 1.31 | -0.019 (0.0003) | 1.74 | -0.019 (0.052) | 1.73 |
|  | z-gynoid | 0.197 (5.83 x 10^-28^) | 0.221 (3.58 x 10^-22^) | 0.078 (NS) | 1.25 | -0.015 (NS) | 3.87 | -0.022 (NS) | 3.68 |
|  | z-leg | 0.226 (1.73 x 10^-24^) | 0.242 (6.27 x 10^-27^) | 0.123 (NS) | 1.23 | 0.031 (NS) | 2.72 | 0.018 (NS) | 2.68 |
|  |  |  |  |  |  |  |  |  |  |
| **Women (n=2,658)** | z-waist | 0.233 (2.86 x 10^-34^) | 0.243 (4.69 x 10^-34^) | 0.110 (2.90 x 10-09) | 1.33 | -0.002 (NS) | 1.74 | 0.006 (NS) | 1.71 |
|  | z-hip | 0.310 (1.06 x 10^-63^) | 0.304 (7.04 x 10^-36^) | 0.139 (NS) | 1.37 | 0.023 (NS) | 2.15 | 0.021 (NS) | 2.11 |
|  | z-android | 0.234 (2.65 x 10^-36^) | 0.255 (1.51 x 10^-38^) | 0.135 (6.97 x 10-12) | 1.27 | 0.013 (NS) | 4.39 | -0.006 (NS) | 4.18 |
|  | z-VAT | 0.144 (1.27 x 10^-14^) | 0.147 (5.66 x 10^-14^) | 0.047 (NS) | 1.24 | -0.069 (0.006) | 1.35 | -0.052 (NS) | 1.38 |
|  | z-gynoid | 0.257 (1.03 x 10^-43^) | 0.260 (2.44 x 10^-40^) | 0.138 (NS) | 1.25 | -0.003 (NS) | 3.25 | -0.009 (NS) | 3.16 |
|  | z-leg | 0.266 (7.63 x 10^-47^) | 0.264 (8.07 x 10^-42^) | 0.149 (NS) | 1.24 | 0.014 (NS) | 2.35 | 0.002 (NS) | 2.38 |
|  | **Leg BMD** |  |  |  |  |  |  |  |  |
| **Men (n=2,097)** | z-waist | 0.398 (1.82 x 10^-47^) | 0.308 (2.04 x 10^-49^) | 0.042 (NS) | 1.42 | -0.018 (NS) | 2.67 | -0.013 (NS) | 2.54 |
|  | z-hip | 0.401 (4.80 x 10^-83^) | 0.364 (1.85 x 10^-65^) | 0.079 (0.00002) | 1.45 | 0.029 (NS) | 2.01 | 0.026 (NS) | 1.97 |
|  | z-android | 0.214 (2.41 x 10^-23^) | 0.243 (6.48 x 10^-31^) | 0.063 (NS) | 1.29 | -0.001 (NS) | **7.11** | -0.005 (NS) | **6.59** |
|  | z-VAT | 0.156 (6.31 x 10^-13^) | 0.193 (6.33 x 10^-20^) | 0.031 (NS) | 1.31 | -0.032 (NS) | 1.74 | -0.017 (NS) | 1.73 |
|  | z-gynoid | 0.232 (3.21 x 10^-27^) | 0.236 (7.18 x 10^-28^) | 0.085 (NS) | 1.25 | 0.010 (NS) | 3.87 | 0.003 (NS) | 3.68 |
|  | z-leg | 0.224 (2.82 x 10^-25^) | 0.222 (2.81 x 10^-25^) | 0.094 (0.00004) | 1.23 | 0.020 (NS) | 2.72 | 0.010 (NS) | 2.68 |
|  |  |  |  |  |  |  |  |  |  |
| **Women (n=2,658)** | z-waist | 0.264 (9.91 x 10^-44^) | 0.287 (1.62 x 10^-31^) | 0.075 (4.71 x 10^-06^) | 1.33 | -0.006 (NS) | 1.74 | 0.003 (NS) | 1.71 |
|  | z-hip | 0.262 (6.39 x 10^-88^) | 0.348 (2.93 x 10^-80^) | 0.107 (NS) | 1.37 | 0.023 (NS) | 2.15 | 0.020 (NS) | 2.11 |
|  | z-android | 0.236 (7.40 x 10^-37^) | 0.281 (1.35 x 10^-20^) | 0.101 (1.47 x 10^-07^) | 1.27 | 0.006 (NS) | 4.39 | 0.002 (NS) | 4.18 |
|  | z-VAT | 0.137 (3.09 x 10^-13^) | 0.169 (1.06 x 10^-17^) | 0.012 (NS) | 1.24 | -0.072 (<0.001) | 1.35 | -0.052 (0.024) | 1.38 |
|  | z-gynoid | 0.268 (1.13 x 10^-47^) | 0.273 (2.52 x 10^-42^) | 0.100 (2.15 x 10^-07^) | 1.25 | -0.002 (NS) | 3.25 | -0.010 (NS) | 3.16 |
|  | z-leg | 0.278 (4.14 x 10^-51^) | 0.275 (3.83 x 10^-49^) | 0.115 (2.97 x 10^-09^) | 1.24 | 0.017 (NS) | 2.35 | 0.003 (NS) | 2.38 |

NS-non significant. Significant p values are set at p<0.001 after Bonferroni correction for multiple testing.

Sβ represents corresponding SD increase in regional BMD with one SD increase in regional fat measured using DXA and anthropometry and total lean mass. Data presented for z-transformed fat and lean mass.

Model 1: adjusted for total lean mass, age, height, smoking status, alcohol intake, physical activity and menopausal status in women.

Model 2: Model 1 + adjusted additionally for total fat mass

Model 3: Model 2 + adjusted additionally for HOMA IR

A VIF of <5 was considered acceptable indicating no collinearity between the adjusted variables.
